# Increasing prevalence of myopia in patients undergoing retinal detachment repair: an 11-year service evaluation

**DOI:** 10.1038/s41433-023-02891-4

**Published:** 2023-12-09

**Authors:** Annegret Dahlmann-Noor, Anton Jaselsky, Chris Whiting, Yvonne Kana, Lyndon da Cruz

**Affiliations:** 1 University College London, London, UK; 2 Moorfields Eye Hospital NHS Foundation Trust, London, UK; 3 NIHR Moorfields BRC, London, UK

**Keywords:** Risk factors, Retinal diseases

Recent national audits indicate that over the past 10–20 years, there has been a substantial increase in surgical retinal detachment (RD) repairs in England and Scotland [1, 2]. Epidemiological studies report that the prevalence of myopia in children and young people has doubled over the past 50 years [3]. Myopia now affects 25–30% of young adults across Europe [4]. With earlier onset and faster and longer progression, more people reach high myopia [5, 6]. Whilst the national vitreoretinal audits discuss myopia as contributing to increasing RD repairs [1, 2] and note that the greatest increase has occurred in men age 50–69 and women age 40–59 [2], they do not systematically collect refractive or biometry data, specifically axial length. Axial elongation typically occurs and progresses during childhood and adolescence, whilst retinal breaks and detachments typically occur in adulthood. To explore whether there has been an increase in the proportion of patients with myopia within those undergoing RD repair over the past 10 years, we carried out a retrospective service evaluation in a large tertiary eye hospital in England.

We summarised the number of hospital episodes from 01/01/2012-30/10/2023, using procedure/OPCS4 codes C79.2, vitrectomy using pars plana approach AND C79.5, internal tamponade of retina using gas, AND (C85.1, retinopexy using cryotherapy OR C81.2, laser photocoagulation of retina for detachment). We then combined these with ICD-10 code “Myopia – H52.1”, and further stratified patients into 5-year age brackets. At our facility, coding for “myopia” is carried out by non-medical staff, based on words mentioned in the medical record, i.e. history and correspondence, and not necessarily based on selection of myopia as diagnosis by a clinician. To determine the accuracy of coding, we ran a prospective service evaluation (registration number 1117). During two weeks at the beginning and end of March 2023, senior nursing staff carried out focimetry of the spectacles of consecutive patients presenting to our vitreoretinal emergency unit. Two months later, we reviewed these records, identifying those who had undergone surgical retinal repairs, and compared the spherical equivalent refraction of the affected eye on focimetry with coding for myopia. Lastly, we reviewed the depth of coding of complex presentations, by reviewing the average number of codes entered per patient episode. The search revealed 20,149 procedures performed between January 2012 and October 2023. A rise in the total number of procedures until 2018 was followed by a plateau, with a temporary reduction in 2020, during COVID-19 pandemic (Fig. 1a). The number of patients under the age of 60 years and those age 60 and over was similar (Fig. 1a). Equally, the number of patients with myopia undergoing these procedures increased, with more patients under the age of 60 (Fig. 1b). The proportion of patients with myopia rose from less than 10% in 2012 to around 25% in 2016, and then to over 40% in 2023, and in the younger age group to over 50% (Fig. 1c).

**Fig. 1:**
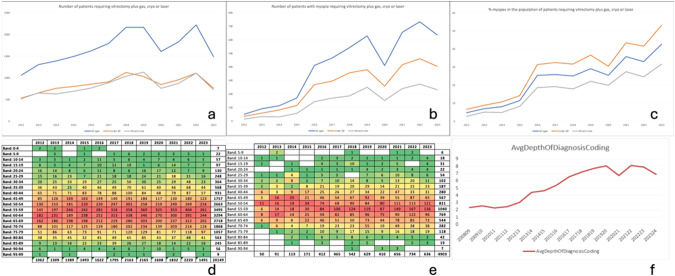
Between 2012 and October 2023, 20,149 procedures (pars plana vitrectomy plus gas with either cryotherapy or laser) were carried out. The absolute number of procedures increased until 2018, with similar numbers of patients under and over the age of 60 years, then reached a plateau, with a temporary reduction in 2020, during COVID-19 pandemic (**a**). Equally, the number of patients with myopia increased, but with more patients under the age of 60 years (**b**). The proportion of patients with myopia rose from around 25% in 2016 to over 40% in 2023, and in the younger age group to over 60% (**c**). Heatmaps show that whilst in 2012 and 2013, the majority of patients were in the age group from 60 to 64 years (red), in 2022 and 2023, the leading age group is five years younger, i.e. 55–59 years (**d**); this is also the most prevalent age group in patients with myopia (**e**). The number of codes registered per patient episode increased over time (**f**). From 2013/14, coding would have been sufficiently accurate for our search.

More detailed analysis of age groups showed that whilst in 2012 and 2013, the majority of patients were in the age group from 60 to 64 years, the now leading age group is five years younger, i.e. 55–59 years (Fig. 1d). This is also the most prevalent age group in patients with myopia (Fig. 1e).

Focimetry data were collected for 41 patients presenting consecutively to the vitreoretinal emergency unit during two weeks in March 2023. Seventeen cases were excluded (14 - no surgical procedure was carried out, one - surgery not for RD, one – duplicate patient, and one - focimetry not carried out for the affected side).

We included 24 records. In 20 eyes, focimetry indicated myopia, and in 11 of these, coding also included myopia. The other 9 were not coded as myopic (37.5%). Of these, one eye was pseudophakic; the fellow-eye was myopic, so the eye with RD may have been myopic prior to insertion of a lens implant. Four focimetries did not show myopic prescriptions; of these 3 were coded correctly (including 2 pseudophakic eyes), and 1 was wrongly coded as myopic (pseudophakic, with myopic fellow eye).

This means that focimetry and coding agreed in 14/24 cases (58.3%), that coding did not include myopia in 9/24 (37.5%) of cases where focimetry indicated it was present (under-reporting), and that it potentially over-reported myopia in 1/24 cases (4.2%).

Analysis of depth-of-coding showed that over time, the clinical coding team recorded higher numbers of diagnoses/procedure codes per patient episode (Fig. 1f). From 2013/14, an average of 4 diagnoses/procedures was used, the minimum required to capture our codes of interest.

Our main finding is that the proportion of myopes in those undergoing RD repair has sharply increased over the past 10, particularly over the past 6 years, to now over 40%. In younger people, i.e. age 60 years and less, people with myopia constitute over 50% of patients.

This work is limited by including a single site, depending on non-specialist documentation of myopia. We attempted to test data quality in a prospective evaluation of focimetry to explore myopia prevalence, but found that this is not a robust marker: many patients had undergone cataract surgery with lens implantation aiming for emmetropia. This may have led to underestimation of myopia prevalence. Concerns about potential over-reporting of myopia based on coding remain unclear. In our evaluation at least, more patients had myopia on focimetry than on coding. Axial length would be a more robust marker of myopia, acknowledging that in cases of macular detachment, this parameter might also be erroneous. A strength is the number of cases, over 20,000. During the observation period, the same electronic record and coding systems have been used, but the practice of vitreoretinal surgery and recording accuracy and methods have changed.

Going forward, we would recommend including refractive error and axial length in national audits of vitreoretinal procedures to facilitate epidemiological and health-economical evaluations of the rising cost of myopia to the NHS.

## Data Availability

Data will be made available upon request.
